# Dali server: structural unification of protein families

**DOI:** 10.1093/nar/gkac387

**Published:** 2022-05-24

**Authors:** Liisa Holm

**Affiliations:** Institute of Biotechnology, Helsinki Institute of Life Sciences, and Organismal and Evolutionary Biology Research Program, Faculty of Biosciences, University of Helsinki, Finland

## Abstract

Protein structure is key to understanding biological function. Structure comparison deciphers deep phylogenies, providing insight into functional conservation and functional shifts during evolution. Until recently, structural coverage of the protein universe was limited by the cost and labour involved in experimental structure determination. Recent breakthroughs in deep learning revolutionized structural bioinformatics by providing accurate structural models of numerous protein families for which no structural information existed. The Dali server for 3D protein structure comparison is widely used by crystallographers to relate new structures to pre-existing ones. Here, we report two most recent upgrades to the web server: (i) the foldomes of key organisms in the AlphaFold Database (version 1) are searchable by Dali, (ii) structural alignments are annotated with protein families. Using these new features, we discovered a novel functionally diverse subgroup within the WRKY/GCM1 clan. This was accomplished by linking the structurally characterized SWI/SNF and NAM families as well as the structural models of the CG-1 family and uncharacterized proteins to the structure of Gti1/Pac2, a previously known member of the WRKY/GCM1 clan. The Dali server is available at http://ekhidna2.biocenter.helsinki.fi/dali. This website is free and open to all users and there is no login requirement.

## Graphical Abstract

**Graphical Abstract:**
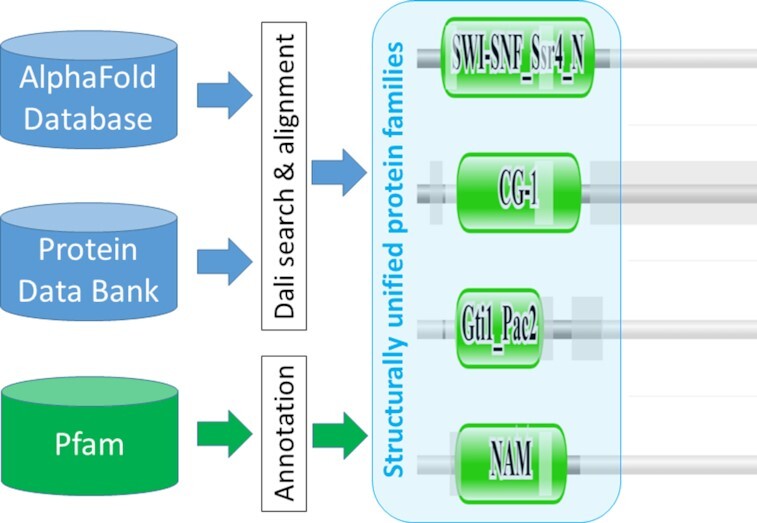
Dali server supports structural searches against the Protein Data Bank and AlphaFold Database and annotates structural alignments with Pfam graphics.

## INTRODUCTION

Current life forms derive from a universal common ancestor that lived four billion years ago (1). The appearance of new molecular functions during much of biological evolution has rested on subtle alteration of pre-existing protein structures (e.g. 2–4). Many protein families have diversified beyond recognition by sequence comparison, their evolutionary relationship being recoverable only with support from structure comparison (5). Recent breakthroughs (6,7) in accurate protein structure prediction dramatically increase the scope of comparative structural studies (8). Using publically available software (https://github.com/sokrypton/ColabFold), structural models can be predicted with confidence for families that do not display any sequence similarity with known protein families. The publically available AlphaFold Database version 1 (AF-DB) covers the Uniprot reference proteomes of human and 20 other key organisms (9). We have imported AF-DB to the Dali server. To our knowledge, this is the first web server to provide sensitive structural searches against AF-DB.

**Figure 1.**
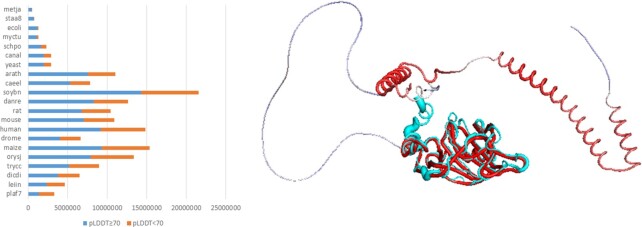
**Left:** Residue-wise proteome coverage by AlphaFold. Residues with pLDDT above 70 are confidently modelled. Species identified by Swissprot short names, sorted by fraction of confidently modelled residues. **Right:** AlphaFold Database model of Ssr4 superimposed onto the crystal structure of the N-terminal domain (7k7v, cyan). The AF-DB model is colored by pLDDT (red: high values, blue: low values). The crystal structure has an N-terminal His tag not present in the natural protein. The structures superimpose with 0.2 Å rmsd over 180 CA atoms. Image rendered by Pymol using superimposed coordinates downloaded from the Dali web server.

AF-DB provides a per-residue confidence metric called pLDDT. pLDDT values above 70 indicate generally good backbone prediction. Lower confidence pLDDT bands correlate with disordered regions (10). They appear as unstructured, wide arcs in the models, which should be ignored when interpreting structural features (Figure 1). Dali alignments concentrate on compact globular domains. With accurate models readily available for the most part of most proteins, structural analysis will become a mainstay in function prediction (e.g. 11–13).

Complete proteomes contain many paralogs which are detectable by standard sequence comparison and have highly conserved structures. The Dali server provides PDB search results filtered to representative subsets at 90%, 50% or 25% sequence identity. As a new feature, we have included the option of Pfam annotations. Pfam annotations cover ∼75% of proteins and ∼49% of residues (14). While many PDB structures represent isolated domains, AF-DB models proteins as a whole (i.e. end-to-end) and long proteins are typically annotated with multiple domains. We show the location of Pfam domains overlaid on structural alignments, making it easy to identify interesting new structural relationships between Pfam families. In this paper, we present a case study where mining AF-DB and using the enhanced analytics led to the structural unification of several protein families.

## MATERIALS AND METHODS

### Inputs and outputs

Dali is a protein structure comparison server based on distance matrix comparison (15). The web server (http://ekhidna2.biocenter.helsinki.fi/dali/) supports searching three databases (the Protein Data Bank (PDB); the PDB25 representative subset of PDB; and the AlphaFold Database version 1) as well as two types of customized structure comparisons (one-against-many pairwise comparison and all-against-all comparison). The web server takes the structure identifier or the 3D coordinates of protein structures as input and returns a list of similar structures, structural alignments and superimposed structures. In addition, the all-against-all comparison returns a structural dendrogram. The web server supports discovery by interactive visualization tools, including a structure viewer and structurally aligned sequence logos (16). Furthermore, it links results to external sequence search (17), functional annotation (18) and family classification resources (14).

### AlphaFold database

AlphaFold Database version 1 models were downloaded from https://alphafold.ebi.ac.uk/. The first release of AF-DB covers the human proteome and the proteomes of twenty other key organisms. The models were given unique four-letter identifiers used internally by Dali. A few models with >200 secondary structure elements were cut into two chains (A and B) in order to fit the array dimensions of the Dali program. Cut points were selected visually in low confidence segments between globular domains. These cut models are listed on our website. Currently, we provide only a hierarchical search (19) against AF-DB, which is limited to one organism at a time.

### Pfam annotations

**Figure 2.**
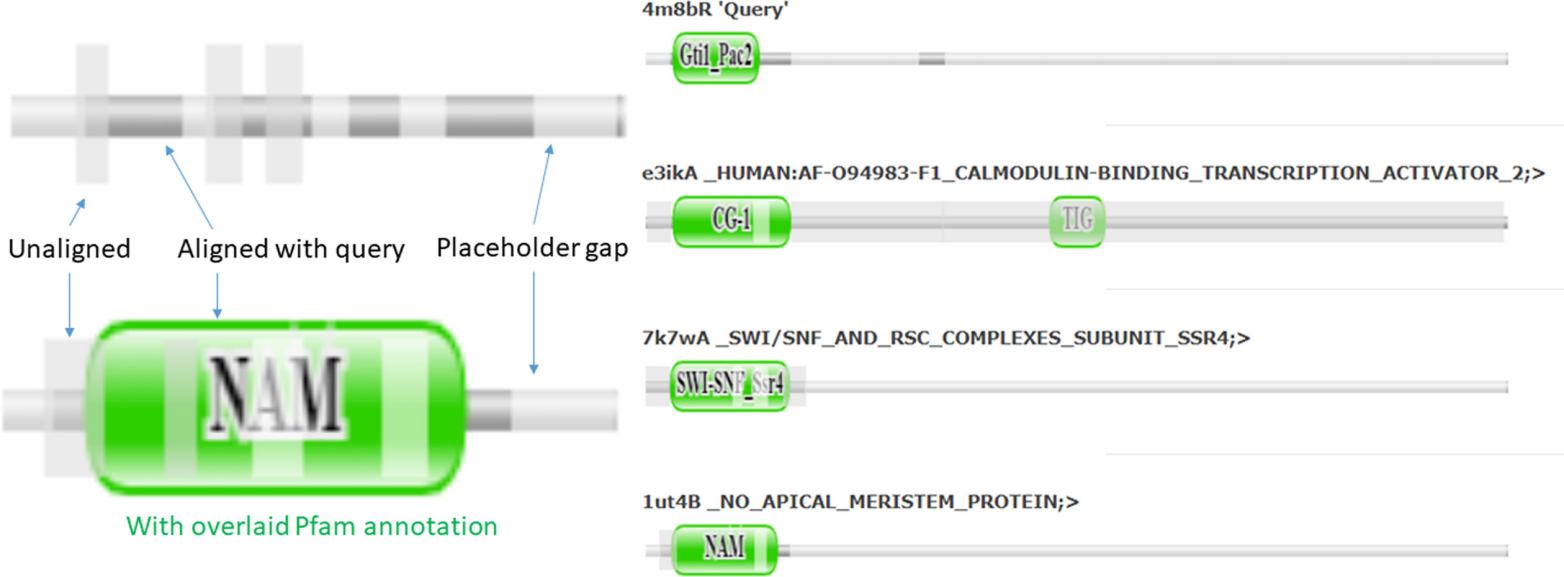
**Left:** Meaning of visual cues in stacked Pfam graphics. Placeholder gaps are introduced in order to show structurally equivalent blocks (dark hue) aligned vertically throughout the stack. Unaligned segments (tall bars) are tucked beneath each other to save space. **Right:** Stacked Pfam graphics of structural neighbors. Pfam annotations are shown when you hover the cursor above a green cartouche. Here, structural alignment unifies the four N-terminal domain families.

Pfam release 35.0 was downloaded from http://ftp.ebi.ac.uk/pub/databases/Pfam/. The location of Pfam families was transferred directly to AlphaFold models, which match Uniprot sequences end-to-end. The location of Pfam families in PDB structures was determined by alignments of sequences extracted from the coordinates to HMMs listed in Pfam's pdbmap table. The annotations are stored internally in an sqlite3 database. The web server displays Pfam annotations using the Pfam domain graphics javascript library (https://pfam-docs.readthedocs.io/en/latest/guide-to-graphics.html). Stacked Pfam graphics show a stylized representation of the query-anchored structural alignment (Figure 2). Placeholder gaps are introduced to show structurally equivalent blocks vertically aligned. Tall bars denote unaligned segments. We reduce clutter by fusing unaligned segments shorter than 10 residues with the adjoining structurally aligned segments. Pfam annotations are available for PDB and AF-DB entries. Annotations for structures uploaded by the user are generated using a hhblits (20) search against Pfam.

## RESULTS

### Novelties

#### Structural search of AlphaFold Database

Users can specify the query structure using Dali's internal structure identifier or upload a PDB formatted file. Dali's internal structure identifiers concatenate a four-letter entry identifier and the chain identifier. Historically, we use the four-letter PDB entry code. Thus, if you find an interesting structure, e.g. at the RCSB site (http://rcsb.org), you can type its PDB identifier into the submission form. AF-DB structures were imported to Dali using our own renaming scheme. For web server users, there are two ways to find out what these internal AF-DB identifiers are: (i) doing a sequence search against AF-DB with the SANSparallel server (17), or (ii) picking the identifiers of interesting matches from a previously done Dali search against the AlphaFold Database. Users of the standalone program can download preprocessed AF-DB data that includes cross-references between Dali and AF-DB/Uniprot. Both pairwise and all-against-all structure comparisons accept sets of mixed PDB and AF-DB identifiers. All structure database searches accept either a PDB or AF-DB identifier as the query structure.

#### Pfam graphics

The PDB contains 186 218 entries at the time of writing. There are many structures for closely related proteins, so the number reduces to 60 862 polypeptide chains using a 90% sequence identity threshold (PDB90). When asking if a newly solved structure is unique, it is useful to filter the results to a subset of 22 130 representatives using a 25% sequence identity threshold (PDB25). AlphaFold Database (version 1) adds 365 198 structural models for 21 proteomes. Some protein families have numerous paralogs, which all show up in the search results. Protein descriptions are not standardized, so eyeballing through a result list does not immediately reveal which relationships are known and which are new and interesting. Pfam (14) family annotations are an invaluable asset in the filtering process. The stacked Pfam graphics show the location of all Pfam domains along the protein sequence, and which domains are in structurally aligned regions. As Pfam comes with textual descriptions of the salient features of each family including literature references, one can efficiently evaluate potential evolutionary discoveries. Pfam annotations were particularly instructive in the case study discussed below.

### Case study

AlphaFold2 successfully modelled CASP14 target T1090 with remarkably low RMSD and a clear gap to the next best predictor (21). T1090 (22) is the N-terminal domain of chromatin remodeling protein Ssr4 from *Schizosaccharomyces pombe*, a member of the SWI/SNF family. The protein was classified in CASP as a free modeling target. However, a Dali search of PDB25, a representative subset of PDB, reveals that T1090 adopts a fold that was, in fact, represented in the public PDB at the time of prediction. The top two hits are a NAC domain (1u4tB; *Z*-score 5.7, 3.3 Å rmsd over 88 CA atoms) (23) and a WOPR domain (4m8bR; *Z*-score 5.6, 3.1 Å rmsd over 100 CA atoms) (24). Pfam annotates these proteins as members of the NAM and Gti1/Pac2 domain families, respectively.

AF-DB searches are currently limited to the proteome of one organism at a time. Dali searches against human and *Arabidopsis* models returned the CG-1 family as the top hit. The best Z-score between T1090 and CG-1 members was 9.8 for an alignment of 106 CA atoms with 2.8 Å rmsd and 22% sequence identity. The next hit was an uncharacterized protein At3g16750 (*Z*-score 6.5, 3.0 Å rmsd over 108 CA atoms), which lacks Pfam annotation. Further hits were *Arabidopsis* models representing the NAM and WRKY families with maximal Z-scores of 6.5 and 4.6, respectively. A Dali search against *Schizosaccharomyces pombe* models returned the self-hit to Ssr4 at the top, followed by two members of the Gti1/Pac2 family (*Z*-score 5.4–5.6, 3.2 Å rmsd over 98–100 CA atoms).

**Figure 3.**
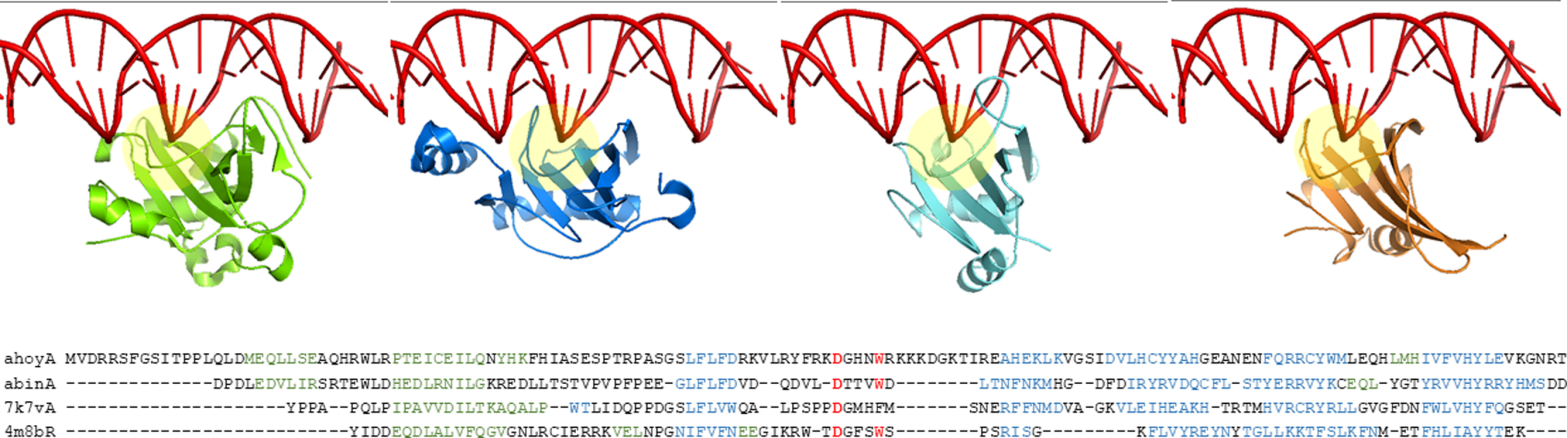
**Top, left to right:** Superimposed structures of 4m8bR (Gti1/Pac2 family) complexed with dsDNA, 7k7vA (22) (residues 1–151) with transplanted dsDNA, CG-1 model (residues 14–139 of ahoyA) with transplanted dsDNA, abinA model (residues 1–133) with transplanted dsDNA. The recognition loop is at the center of the image (yellow highlight). Images rendered by Pymol using superimposed coordinates downloaded from the Dali web server. **Bottom:** Stacked structural alignment anchored on ahoyA N-terminal domain (adapted from web server output). Structurally unaligned insertions relative to ahoyA are omitted. Helices are green, beta strands are blue. The red DxxxW motif stabilizes the recognition loop in 4m8bR (24).

Visualization of the 3D superimposition and structural alignment of family representatives (Table 1) showed that T1090, CG-1, At3g16750, Gti1/Pac2 and NAM share a common fold with αβββββ topology and have an open face where DNA binds to Gti1/Pac2 (Figure 3). The loop joining the edge strands of the beta sheet is involved in sequence-specific DNA binding in Gti1/Pac2 (24). Residues stabilizing the recognition loop are conserved in CG-1. The structural model of CG-1 also makes extensive contacts with the major groove. Sequence-specific DNA binding has been experimentally demonstrated for CG-1 (25). Electrostatic potential calculations (https://pdbj.org/eF-surf/top.do) do not show strong signal for DNA binding by T1090 (22) or At3g16750 (data not shown). Indeed, At3g16750 has a negatively charged tail domain which should repel nucleic acids. The recognition loop is not conserved in the NAM family, which binds dsDNA in a tilted orientation (26) compared to Gti1/Pac2.

**Table 1.** Protein identifiers. New findings are bold

PDB	Dali	AlphaFold database	Pfam	Pfam short name	Other	Description
4m8bR	evi0A	YEAST:AF-P38867-F1	PF09729	Gti1/Pac2	Pfam: CL0274	WHITE-OPAQUE REGULATOR 1
n.a.	ahoyA	ARATH:AF-Q9FY74-F1	PF03859	CG-1	**joins CL0274**	Calmodulin-binding transcription activator 1
7k7vA	gugsA	SCHPO:AF-Q9P7Y0-F1	PF08549	SWI-SNF_Ssr4_N	CASP: T1090 **joins CL0274**	SWI/SNF and RSC complexes subunit ssr4 N-terminal
n.a.	abinA	ARATH:AF-Q9LUQ8-F1	n.a.	n.a.	TAIR: At3g16750 **joins CL0274**	UNCHARACTERIZED PROTEIN
1ut4B, 3swmA	akgpA	ARATH:AF-Q9C932-F1	PF02365	NAM	**joins CL0274**	NAC DOMAIN-CONTAINING PROTEIN 19

In conclusion, although structural resemblance between T1090, CG-1, At3g16750 and the sequence-specific DNA binding domains Gti1/Pac2 was found, such a role is not supported in either T1090 or At3g16750. Interestingly, the last two families have a restricted phylogenetic distribution in fungi and rosids, respectively, suggesting they may be examples of neofunctionalization. Based on structural similarities and/or a general involvement in gene regulation, we place T1090, CG-1, At3g16750, Gti1/Pac2 and NAM in a common superfamily. Gti1/Pac2 is already classified in Pfam as a member of the WRKY/GCM1 clan (CL0274; (27)) which includes seven families of DNA-binding domains. The link between NAM and WRKY/GCM1 was noted by the crystallographers (26) but is not recorded in Pfam 35.0. The common core of the WRKY/GCM1 clan is reduced to a four-stranded beta sheet, so the five families mentioned above form a tighter subgroup within this clan sharing a larger common core. Dali Tutorial 2022 (Supplement) gives a step by step description of the path to discovery.

### Comparison to existing methods

Dali structure comparison has been shown to outperform other methods in the sensitivity of detecting similar folds in the twilight or midnight zones of sequence similarity (15,19,28). In principle, searching the AlphaFold Database is no different from searching the Protein Data Bank. However, the sheer volume of the AlphaFold Database is a practical obstacle and we are not aware of support for AF-DB in other traditional structure database search servers (29–31) or in structural classifications (32–34). To our knowledge, apart from the Dali server, the only server that supports structural searches against both the PDB and AF-DB databases is Foldseek (https://search.foldseek.com/search). FoldSeek transforms structures to a 1D representation using an alphabet of 3D interactions (3Di) so that sequence alignment techniques can be used. Foldseek is very fast but it is clearly less sensitive than Dali at detecting superfamily and fold level similarities (28). As a concrete example, Foldseek in 3Di mode returned 73 PDB25 representatives as similar to 7k7vA. We re-scored these pairwise structural alignments with respect to the Dali *Z*-score: the top three hits ranked 4th, 15th and 20^th^ in the results of a systematic Dali search against PDB25. The self-hit to the query ranked 1st. The discoveries in our case study involved the 2^nd^ and 3^rd^ Dali hits, which were completely missed by Foldseek.

## DISCUSSION

Usage of the Dali server rose sharply after the release of AF-DB in July 2021, increasing queueing times for database searches. Dali performs sensitive but slow searches. Due to the finite capacity of the server, bulk analyses of large sets of queries should be processed locally using the standalone version of DaliLite. The first release of AF-DB is static and pre-processed data is available for download. The PDB is updated weekly, and standalone users must maintain their own copy of the data. The second and third release of AlphaFold Database have increased the number of models close to a million (9). Currently, AF-DB is searched using a hierarchical strategy, which systematically scans a representative subset at 70% sequence identity and expands comparisons to homologs identified by sequence comparison. The Dali server applies a heuristic strategy to search the PDB exploiting a network of known structural similarities that has been built up incrementally for decades (35). A cascade of fast to slow-and-sensitive comparison methods are applied to find entry points to the network, after which the search progresses by ‘walking’ to known structural neighbors of already found hits. This heuristic search strategy scales better to large databases than the hierarchical search (19). We are in the process of building up a knowledge base to speed up searches against AF-DB.

Our case study added three more Pfam families to the WRKY-GCM1 clan (CL0274), namely CG-1, NAM, SWI/SNF, and a small family represented by At3g16750. Apparent functional shifts have happened in the evolution of this superfamily. There are many examples of the phenomenon; for example, dsDNA binding has evolved in some branches of the glucosyltransferase 1 (GT1) superfamily (36,37). The starting point of the study, SWI/SNF, was accurately modelled from sequence by AlphaFold. CG-1 and At3g1675 were modelled by AlphaFold and still lack experimental structures, making them interesting targets for structural genomics.

## DATA AVAILABILITY

The Dali server is available from our website http://ekhidna2.biocenter.helsinki.fi/dali. It runs DaliLite.v5 (19). DaliLite.v5 is an open source software, which can be downloaded to run as a standalone. Preprocessed AlphaFold Database (version 1) data for running DaliLite locally can be downloaded from our website.

## Supplementary Material

gkac387_Supplemental_File
