# Supplementary material for: Dali server: structural unification of protein families
Source: Nucleic Acids Res. 2022 May 24;50(W1):W210–5. doi: 10.1093/nar/gkac387 (PMC9252788; doi:10.1093/nar/gkac387)
Supplement: gkac387_Supplemental_File [file gkac387_supplemental_file.pdf]

Dali Tutorial 2022: New features

Table of contents

What's new?

Introduction

Case study: CASP target T1090

1. Finding structures for your sequence [*new features*]
2. Searching known structures in the Protein Data Bank
3. Pfam annotations [*new features*]
4. Searching AlphaFold Database (version 1) [*new features*]
5. Putting it all together: structural dendrogram and sequence signatures
6. Conclusion

Box 1: Tips for discovery

What's new?

1) Support for AlphaFold database (version 1)

2) Possibility to generate stacked Pfam graphics, which is a useful tool to filter out redundant hits such as paralogs in AlphaFold database

3) Web-server support to interactively visualize and link results to external sequence search, functional annotation, and family classification resources.

Introduction

Dali (http://ekhidna2.biocenter.helsinki.fi/dali/) is a protein structure comparison server based on distance matrix comparison (https://onlinelibrary.wiley.com/doi/full/10.1002/pro.3749). In favourable cases, structure comparison can reveal distant evolutionary relationships not seen by sequence comparison. The web server supports searches in three databases [Protein Data Bank (PDB); PDB25, a representative subset of PDB; AlphaFold (version 1)] and enables two customised types of structure comparisons: i) pairwise structure comparison to one query structure and ii) all against all structure comparison.

The server takes the 3D coordinates of protein structures as input and returns a list of similar structures, structural alignments and superimposed structures. The all against all comparison also returns a structural dendrogram. The results are linked to sequence search and function prediction servers. *Dali tutorial 2016* explains the web interface of the Dali server using live examples. It is still fully valid. This update, *Dali tutorial 2022,* explains more recently added features: enhanced visualization using stacked Pfam cartoons, and support for the AlphaFold Database. These features are illustrated with a case study (Table 1). Gray boxes mark exercises that you can try out interactively in the web server.

Table 1: Cross-mapped identifiers of proteins discussed in this tutorial. New findings highlighted in yellow.

PDB	Dali	AlphaFold Database	Pfam	Pfam short name	Other	Description
4m8bR	evi0A	YEAST:AF-P38867-F1	PF09729	Gt1/Pac2	Pfam: CL0274	WHITE-OPAQUE REGULATOR 1
n.a.	ahoyA	ARATH:AF-Q9FY74-F1	PF03859	CG-1	joins CL0274	Calmodulin-binding transcription activator 1
7k7vA	gugsA	SCHPO:AF-Q9P7Y0-F1	PF08549	SWI-SNF_Ssr4_N	CASP: T1090 joins CL0274	SWI/SNF and RSC complexes subunit ssr4 N-terminal
n.a.	abinA	ARATH:AF-Q9LUQ8-F1	n.a.	n.a.	TAIR: At3g16750 joins CL0274	UNCHARACTERIZED PROTEIN
1ut4B, 3swmA	akgpA	ARATH:AF-Q9C932-F1	PF02365	NAM	joins CL0274	NAC DOMAIN-CONTAINING PROTEIN 19
4ywzA	gvtqA	STAA8:AF-Q2G2U4-F1	n.a.	n.a.		SENSOR PROTEIN KINASE WALK
4i90A	esidA	ECOLI :AF-P52129-F1	PF03108	DBD-Trp-Mut	Pfam: CL0274	mRNA endoribonuclease toxin LS
2rprA	e1hrA	HUMAN: AF-Q4VC44-F1	PF04500	FLYWCH	Pfam: CL0274	FLYWCH-type zinc finger-containing protein 1
2lexA	ac70A	ARATH: AF-Q9XI90-F1	PF3106	WRKY	Pfam: CL0274	Probable WRKY transcription factor 4
4lmgA	ewieA	YEAST: AF-Q08957-F1	PF08731	AFT	Pfam: CL0274	Iron-regulated transcriptional activator AFT2
1odhA	gcg9A	MOUSE:AF-P70348-F1	PF03615	GCM	Pfam: CL0274	Chorion-specific transcription factor GCMa
n.a.	h0rrA	SOYBN :AF-A0A0R0G5C4-F1	PF03101	FAR1	Pfam: CL0274	FAR1 domain-containing protein

Case study: CASP target T1090

CASP is a blind structure competition organized every two years (https://predictioncenter.org/casp14/). We study CASP14 target T1090. The target is chromatin remodeling protein Ssr4 from *Schizosaccharomyces pombe*. The protein was classified in CASP as a free modeling target, implying that template based modeling was not possible. The crystallographers wrote (https://www.ncbi.nlm.nih.gov/pmc/articles/PMC7716260/): "PDBeFold (Krissinel & Henrick, 2004) was used to compare the Ssr4 structure with all other structures in the PDB. … This suggests that this domain of SSR4 adopts a fold that is not currently represented in the public PDB. For this reason it was submitted to CASP14, with the results of the modelling to be presented in the future." AlphaFold2 predicted the structure with remarkably low RMSD and a clear gap to the next best predictor (https://onlinelibrary.wiley.com/doi/full/10.1002/prot.26172).

1. Finding structures for your protein [*new features*]

First, we use the sequence to identify the corresponding PDB and AF-DB models. Paste the FASTA formatted sequence (https://predictioncenter.org/casp14/target.cgi?id=130&view=all) to the SANSparallel server at http://ekhidna2.biocenter.helsinki.fi/cgi-bin/daliviewer/call_sans.cgi. Select the searched database using the radio buttons. We learn that the best PDB match is *7k7vA* and the best AlphaFold Database match is *gugsA* (Figure 1).

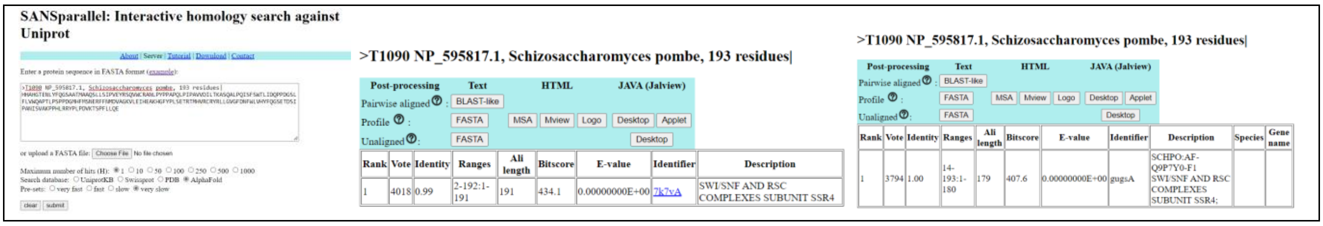

Figure 1. Interactive homology search using SANSparallel: a submission example (left) and outputs from searches in the PDB (middle) and AlphaFold Database (right).

Dali internally uses four-letter identifiers for the PDB entry plus chain identifier. PDB identifiers are inherited from the Protein Data Bank and start with a number 1-9. Dali's internal identifiers for AlphaFold Database (AF-DB) models start with a letter a-h. The original AF-DB identifier can be recovered from the description field (cf. Figure 1).

Second, do a pairwise Dali alignment of *gugsA* and *7k7vA* to verify the high accuracy of the model predicted by AlphaFold2 with 0.2 A RMSD over 192 residues (Figure 2). Important to note that the PDB structure of Ssr4 was released after the CASP14 competition.

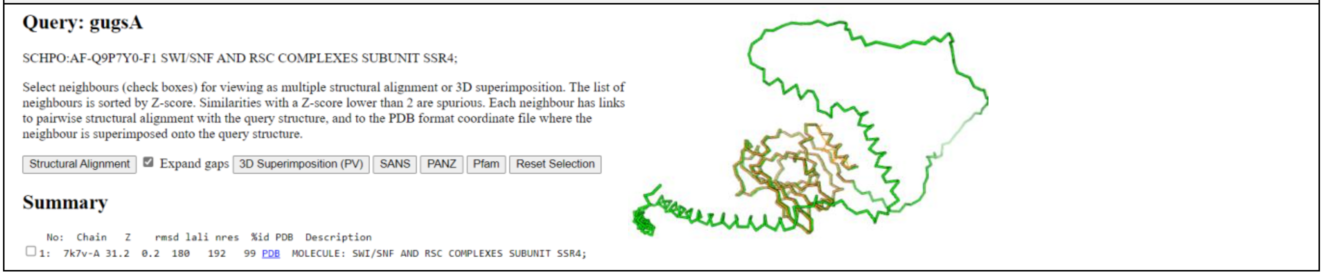

Figure 2. Left: Summary statistics of pairwise alignment of AlphaFold2 model and experimentally determined structure of CASP14 target T1090. Right: structural superimposition of PDB structure 7k7vA (yellow) and AF-DB model gugsA (green).

AlphaFold generates end-to-end models of the whole protein sequence. In the present case, only the N-terminal domain of *gugsA* is modelled as a compact domain. The C-terminal domain is not present in the crystal structure. The crystal structure has an artificial N-terminal His tag, which is naturally not present in the AF-DB model. Subsequent analysis will focus on the N-terminal domain of Ssr4, so we use 7k7vA as query instead of the AF-DB model.

2. Searching known structures in the Protein Data Bank

Next, we did a PDB search for structural neighbors of 7k7vA. For initial screening, it is useful to filter redundant hits by inspecting the top hits in the PDB25 subset (Figure 3-left). The stacked structural alignment shows the extent of the common core (Figure 3-right). The top row shows the query sequence. Structurally equivalent segments from the other structures are mapped onto it. We see that most structures match the beta strands, but the alpha helix preceding the beta strands is present in only three structural neighbors. We'll select these three (1ut4B, 3m8bR, 4ywzA) for further comparison with 7k7vA.

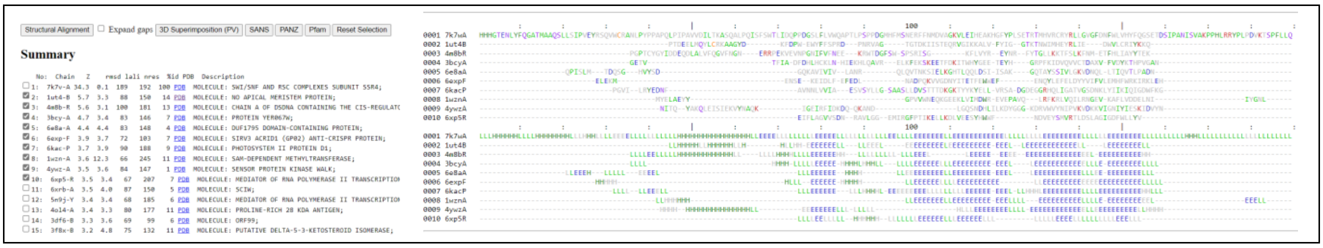

Figure 3. Left: summary list of top hits (PDB25 subset) from PDB search. Right: stacked alignment of selected hits (bottom, upper part shows amino acid sequences, lower part shows secondary structures).

Do a pairwise comparison of 7k7vA (first structure) against 1ut4B, 4m8bR and 4ywzA (second structures) using the pairwise comparison option of the Dali server. Pairwise comparisons are done interactively, whereas there may be a long wait in the queue for PDB searches. You'll get a summary list similar to Figure 3, only shorter. Select all structures and click the *3D Superimposition (PV)* button. This opens a new the Daliviewer window. Inspect the structural overlap between the structures. Hide all structures except the query and color it by Structure Conservation. The common core (blue) forms a compact globule (Figure 4).

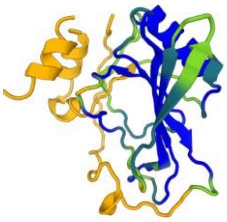

Figure 4. Structural conservation mapped onto 7k7vA. Highly conserved regions are blue.

Click on the PDB link in the summary list and check the HEADER record 1ut4B and 4m8bR. These entries were released by PDB well before CASP14. *(Hint: The coordinates are transformed to superimpose with the query structure. Download them to generate publication-quality graphics in PyMol.)*

1ut4B and 4m8bR were the top hits in the PDB search. The Z-scores are moderate (5.6-5.7) but the common core covers practically all secondary structure elements with an RMSD 3.1-3.3 A over 88-100 Cα atoms. We conclude that contrary to its classification as a free modeling target in CASP, T1090 did not represent a novel fold but suitable templates were present in the PDB.

Continuing in the Daliviewer window, check the *Load whole structures* box at the bottom and check *show other chains of* All. Now you see the DNA molecule complexed with 4mb8R; it is clearest in cartoon

representation (Figure 5). The superimposition allows you to visualize a transplantation of the bound DNA to the other structures. Toggle structures on/off and verify that 7k7vA and 1ut4B have open space where the DNA binds, whereas 4ywzA has an extra helix that blocks the DNA binding site.

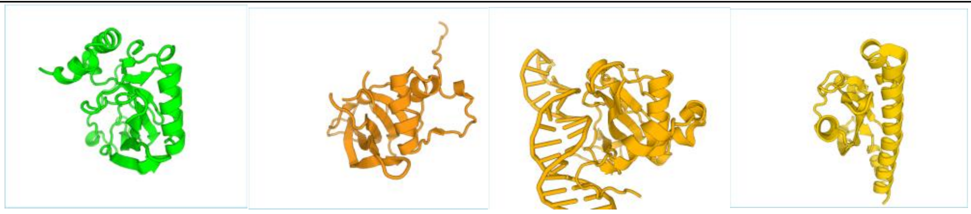

Figure 5. Superimposed structures side by side. From left to right: 7k7vA, 1ut4B, 4m8bR, 4ywzA.

4ywzA had a relatively low Z-score in the PDB search and has a different architecture. We'll drop 4ywzA from further consideration and pursue the hypothesis that T1090 shares the DNA binding function with 4m8bR and 1ut4B.

The loop that connects the edge strands of the beta sheet is structurally conserved between the remaining structures. It is the DNA recognition loop in 4m8bR, fitting into the major groove of dsDNA. We observe that this loop is disordered in 1ut4B, which causes chain breaks in the PDB structure. There are multiple PDB structures for this NAC-domain containing protein 19 (https://www.uniprot.org/uniprot/Q9C932). Some of these structures are complexed with DNA, e.g. PDB entry 3swm. However, they too have disordered loops. Recalling that AlphaFold generates end-to-end models, we will see how these loops are modelled by AlphaFold and whether they interfere with DNA binding. Dali's internal identifier for the AF-DB model is *akgpA*.

Make a new pairwise alignment of 7k7vA (first structure) against akgpA, 1ut4B, 3swmA, 3swmB, 3swmC, 3swmD and 4mb8R (second structures). Verify in the structural alignment view that akgpA models loops missing from the 3swm chains. Inspect the contacts to DNA.

3. Pfam annotations [*new features*]

Next, we study what is known about the biological functions of our proteins of interest. Here, we exploit information gathered in the Pfam database.

Return to the summary page, select all hits and click the *Pfam* button. The Pfam graphics show that the hits belong to different protein families (Figure 6). Hover the cursor above a green cartouche for more information on the domain family. Read the documentation of the families at the Pfam website, e.g. https://pfam.xfam.org/family/PF08549. Substitute the Pfam identifier PFxxxxx for the other families.

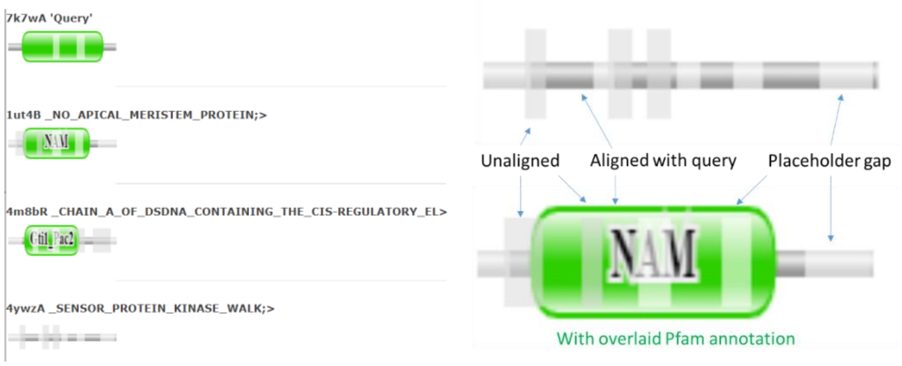

Figure 6. Left: Stacked Pfam graphics of structural neighbors. Right: Meaning of visual cues. Placeholder gaps are introduced in order to show structurally equivalent blocks aligned vertically throughout the stack.

The Pfam view gives a graphical overview of the structural alignment, adding Pfam annotations. The members of a Pfam family share significant sequence similarity, modelled by Hidden Markov models (HMMs). Pfam annotations were taken from Pfam 35.0. Pfam annotations cover ~75% of proteins and ~49% of residues (https://xfam.wordpress.com/2021/11/19/pfam-35-0-is-released/). Large families are well covered by Pfam (https://academic.oup.com/nar/article/33/suppl_1/D188/2505389), smaller families are collected in Pfam-B but not included in our Pfam annotation database. Pfam is derived from proteins in the Uniprot database. Pfam annotations are available for PDB structures (in Pfam's pdbmap table) and AF-DB models (which are based on Uniprot sequences). User may upload their own structures to the Dali server, but these will not have associated Pfam annotation. Since Pfam releases have an interval of a year, annotations will also be missing for the most recent PDB structures.

It is common for distinct Pfam families to share structural similarity, either by common descent (homology) or by physical convergence. In our example (Figure 4), we found that three domain families share a common fold: SWI/SNF and RSC complexes subunit Ssr4 N-terminal (PF08549), no apical meristem (NAM) protein (PF02365), and the Gt1/Pac2 family (PF09729). Intriguingly, they are all involved in gene regulation. Pfam places the Gt1/Pac2 family in the WRKY-GCM1 clan (CL0274). Pfam clans unify remote homologs. No clan is assigned to the other two families. WRKY and GCM1 are metal chelating DNA-binding domains (DBD) which share a four-stranded fold (http://www.ncbi.nlm.nih.gov/pubmed/17130173). Our families share a larger core consisting of five strands and a helix (Figure 2).

4. Searching AlphaFold Database (version 1) [*new features*]

So far, we have done a PDB search and identified known structures with the same fold as T1090. Let's move on to check if AF-DB presents us with more potential members of the emerging superfamily of DNA-binding domain (Box 1). PDB searches (*PDB search* and *PDB25 search* tabs) and AF-DB searches (*AF-DB search* tab) are kept separate in the Dali server. PDB contains experimental structures, whereas AF-DB contains (remarkably accurate) predictions.

AF-DB searches in Dali are currently limited to one model organism at a time. You choose from 21 model organisms, including human. AF-DB searches have long queueing times. Therefore, we show below the top hits from precomputed searches of 7k7vA against HUMAN (Figure 7). Visual inspection shows that members of the CG-1 family (PF03859), represented by e.g. e3ikA, resemble the DNA-binding domains. A search against *Arabidopsis* again shows CG-1 as the top hit, an uncharacterized protein At3g16750 at rank 6, and the previously picked up NAM family as the next best hits (Figure 8). Also WRKY domains match the beta sheet. A four-stranded beta sheet is a common structural motif, so it is noteworthy that WRKY domains match *better* than other beta sheet proteins. A search against fission yeast recovers the query (Ssr4) and two paralogs of the Gt1/Pac2 family, already known members of our superfamily (Figure 9). Other top hits did not pass the secondary structure filtering step.

```
# Job: HUMAN
# Query: 7k7vA
# No:  Chain   Z    rmsd lali nres  %id PDB  Description
   1:  e3ik-A  9.6  3.4  115  1202   18   HUMAN:AF-O94983-F1 CALMODULIN-BINDING TRANSCRIPTION ACTIVATOR 2;
   2:  fckc-A  3.7  4.6   91  1070    4   HUMAN:AF-O15063-F1 GRANULE ASSOCIATED RAC AND RHOG EFFECTOR PROTEIN
   3:  e6yp-A  3.5  4.1   84   626    5   HUMAN:AF-Q9GZY0-F1 NUCLEAR RNA EXPORT FACTOR 2;
   4:  e1v1-A  3.4  5.3   82  1724    9   HUMAN:AF-Q9UKK3-F1 PROTEIN MONO-ADP-RIBOSYLTRANSFERASE PARP4;
   5:  fev8-A  3.1  4.4  124  1673   17   HUMAN:AF-Q9Y6Y1-F1 CALMODULIN-BINDING TRANSCRIPTION ACTIVATOR 1;
   6:  fdbh-A  3.1  3.4   86   186    7   HUMAN:AF-Q9HD47-F1 RAN GUANINE NUCLEOTIDE RELEASE FACTOR;
   7:  e9iu-A  3.1 17.0   74   608    7   HUMAN:AF-Q86X55-F1 HISTONE-ARGININE METHYLTRANSFERASE CARM1;
   8:  e2gj-A  3.0  3.5   75   140    7   HUMAN:AF-P35080-F1 PROFILIN-2;
   9:  e62h-A  2.9  4.2   76  1332    7   HUMAN:AF-O95163-F1 ELONGATOR COMPLEX PROTEIN 1;
  10:  fa7h-A  2.8  4.1   80  1400    5   HUMAN:AF-Q5T011-F10 KICSTOR COMPLEX PROTEIN SZT2;
```

Figure 7. AF-DB search result against the human proteome. CG-1 family (PF03859) members are bold.

```
# Job: ARATH
# Query: 7k7vA
# No:  Chain   Z    rmsd lali nres  %id PDB  Description
   1:  aulq-A  9.8  2.8  106   923   22   ARATH:AF-O23463-F1 CALMODULIN-BINDING TRANSCRIPTION ACTIVATOR 5;
   2:  ahoy-A  9.7  2.8  105  1007   22   ARATH:AF-Q9FY74-F1 CALMODULIN-BINDING TRANSCRIPTION ACTIVATOR 1;
   3:  akf2-A  9.4  2.7  104  1032   23   ARATH:AF-Q8GSA7-F1 CALMODULIN-BINDING TRANSCRIPTION ACTIVATOR 3;
   4:  aote-A  9.2  2.7  104  1050   24   ARATH:AF-Q6NPP4-F1 CALMODULIN-BINDING TRANSCRIPTION ACTIVATOR 2;
   5:  aanf-A  8.9  3.0  106  1016   29   ARATH:AF-Q9FYG2-F1 CALMODULIN-BINDING TRANSCRIPTION ACTIVATOR 4;
   6:  abin-A  6.5  2.8  103   194   17   ARATH:AF-Q9LUQ8-F1 UNCHARACTERIZED PROTEIN;
   7:  apry-A  6.5  5.5  112   322   10   ARATH:AF-O80752-F1 APICAL MERISTEM FORMATION PROTEIN-RELATED;
   8:  ah0e-A  6.3  3.4   99   276    9   ARATH:AF-F4IME8-F1 NAC DOMAIN CONTAINING PROTEIN 36;
   9:  aqv9-A  6.2  3.3  103   320   13   ARATH:AF-O80756-F1 NAC DOMAIN CONTAINING PROTEIN 24;
  10:  aki9-A  6.1  3.2   97   175   13   ARATH:AF-Q1ECJ5-F1 AT1G60240;
// snip //
  75:  aajg-A  4.8  4.2  107   469   11   ARATH:AF-Q9SCK6-F1 NAC DOMAIN-CONTAINING PROTEIN 62;
  76:  ac99-A  4.8  3.3   99   528   14   ARATH:AF-F4IED2-F1 NAC DOMAIN-CONTAINING PROTEIN 13;
  77:  arkn-A  4.8  4.6   79   274   10   ARATH:AF-Q9FHR7-F1 PROBABLE WRKY TRANSCRIPTION FACTOR 49;
  78:  asoz-A  4.8  4.5   89   195    8   ARATH:AF-Q8VWQ4-F1 PROBABLE WRKY TRANSCRIPTION FACTOR 56;
  79:  addy-A  4.7  3.4   98   212   13   ARATH:AF-Q9FLM0-F1 AT5G41090;
  80:  aqg2-A  4.6  3.0   94   451   14   ARATH:AF-Q9SQY0-F1 NAC DOMAIN CONTAINING PROTEIN 52;
  81:  am7i-A  4.6  3.6   97   303   11   ARATH:AF-Q9LSI4-F1 NAC (NO APICAL MERISTEM) DOMAIN TRANSCRIPTIONAL R
  82:  amrf-A  4.6  4.7   81   337   10   ARATH:AF-O22900-F1 WRKY TRANSCRIPTION FACTOR 23;
  83:  ac8d-A  4.6  3.9   84   374    6   ARATH:AF-Q9C9F0-F1 PROBABLE WRKY TRANSCRIPTION FACTOR 9;
  84:  adw2-A  4.6  4.9   87   557    6   ARATH:AF-Q93WV0-F1 PROBABLE WRKY TRANSCRIPTION FACTOR 20;
```

Figure 8. AF-DB search result against the Arabidopsis proteome. CG-1 (bold), NAM and WRKY domain families populate the list. abinA (rank 6) is not classified in Pfam.

```
# Job: SCHPO
# Query: 7k7vA
# No:  Chain   Z    rmsd lali nres  %id PDB  Description
   1:  gugs-A 31.2  0.2  180   395   99   SCHPO:AF-Q9P7Y0-F1 SWI/SNF AND RSC COMPLEXES SUBUNIT SSR4;
   2:  gshl-A  5.6  3.2   98   720   12   SCHPO:AF-O14367-F1 GLUCONATE TRANSPORT INDUCER 1;
   3:  gs5d-A  5.4  3.2  100   235    9   SCHPO:AF-Q10294-F1 CAMP-INDEPENDENT REGULATORY PROTEIN PAC2;
   4:  guob-A  2.9  4.1   83   190    5   SCHPO:AF-O75002-F1 NUCLEAR IMPORT PROTEIN MOG1;
   5:  gtiw-A  2.9  4.1   66   673    5   SCHPO:AF-O13650-F1 PUTATIVE TRANSCRIPTION FACTOR TAU SUBUNIT SFC9;
   6:  gq17-A  2.8  5.5   76  1611    4   SCHPO:AF-O42854-F1 SH3 DOMAIN-CONTAINING PROTEIN C23A1.17;
   7:  gryg-A  2.5  4.1   77   434    9   SCHPO:AF-O94260-F1 PUTATIVE G3BP-LIKE PROTEIN;
   8:  gryy-A  2.5  4.2   81   832    5   SCHPO:AF-Q7Z9H9-F1 POLYPHOSPHOINOSITIDE PHOSPHATASE;
   9:  gt0x-A  2.5  4.0   69   141    4   SCHPO:AF-Q9USW0-F1 UNCHARACTERIZED PROTEIN C21B10.02;
  10:  grce-A  2.4  4.5   75  1004    5   SCHPO:AF-Q10408-F1 UNCHARACTERIZED PROTEIN C1F3.03;
```

Figure 9. AF-DB search result against the fission yeast proteome. Gt1/Pac2 family members are bold.

5. Putting it all together: structural dendrogram and sequence signatures

We performed an all-against-all comparison using representatives of old and new clan members (Figure 10). For this analysis, we used cropped AF-DB models and a local DaliLite installation (see *Download* tab on Dali server website). Cropped AF-DB models retain only residues that are confidently modelled (pLDDT>70). Cropping removes non-compact loops in AF-DB models, like the C-terminal part of T1090 (see Figure 2), which add spurious equivalences to Dali alignments that destroy the 3-D superimposition.

Copy-paste the resulting newick tree to iToL (https://itol.embl.de/):

```
(((((((ahoyA_PF03959_CG-
1:38.5,abinA_n.a.:14.1):4.6,gugsA_PF08549_SWI/SNF:25.7):1.6,evi0A_PF09729_Gt1/Pac2:25.2):0.4,akgpA_PF02365_NAM:22.1):2.2,(gcg9A_PF036
15_GCM:23.5,ac70A_PF03106_WRKY:17.2):1.4):1.45714285714286,(((ewieA_PF08731_AFT:16.9,h0rrA_PF03101_FAR1:14.3):3.3,esidA_PF03108_DBD-
Trp-Mut:39.8):0.966666666666667,e1hrA_PF04500_FLYWCH:36.0666666666667):0.090476190476191):3.24285714285714,0.1:0):0.1);
```

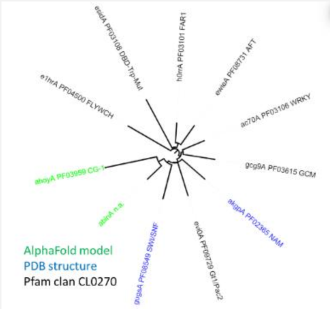

abinA n.a.
ac70A PF03106 WRKY
ahoyA PF03959 CG-1
akgpA PF02365 NAM
e1hrA PF04500 FLYWCH
esidA PF03108 DBD-Trp-Mut
evi0A PF09729 Gt1/Pac2
ewieA PF08731 AFT
gcg9A PF03615 GCM
gugsA PF08549 SWI/SNF
h0rrA PF03101 FAR1

e1hrA	39.5	2.4	3.6	4.3	5.3	3.5	0.1	0.1	**0.1**	0.1	0.1
esidA	2.4	44.2	4.3	4.5	4.3	3.3	0.1	0.1	**2.3**	2.6	2.7
h0rrA	3.6	4.3	22	7.7	5.1	3	0.1	0.1	**0.1**	2.5	2.7
ewieA	4.3	4.5	7.7	24.6	6.6	4	2.4	2.1	**2.3**	2.9	3.1
ac70A	5.3	4.3	5.1	6.6	23.4	6.2	4.2	3.6	**4.3**	5.3	5.5
gcg9A	3.5	3.3	3	4	6.2	29.7	3.3	4.9	**3.1**	5.6	7.1
akgpA	0.1	0.1	0.1	2.4	4.2	3.3	29.1	3.4	**5.5**	5.5	7
evi0A	0.1	0.1	0.1	2.1	3.6	4.9	3.4	32.6	**5.2**	6.5	7.4
gugsA	**0.1**	**2.3**	**0.1**	**2.3**	**4.3**	**3.1**	**5.5**	**5.2**	**34.7**	**7.5**	**9**
abinA	0.1	2.6	2.5	2.9	5.3	5.6	5.5	6.5	**7.5**	27.7	13.6
ahoyA	0.1	2.7	2.7	3.1	5.5	7.1	7	7.4	**9**	13.6	52.1

Figure 10. Left: Structural dendrogram of extended WRKY-like clan using AF-DB models. **Middle:** internal Dali-identifiers of family representatives. **Right:** Z-score matrix for cropped AF-DB models. Our study object T1090/gugsA/SWI-SNF is bold. Z-scores between 2 and 5 are pink, Z-scores between 5 and 14 are yellow.

The crystal structure of SWI/SNF and the AF-DB models of CG-1 and unclassified abinA share strong structural similarity and form a subgroup with the Gt1/Pac2 family. CG-1 is a specific DNA-binding protein ([CG-1, a parsley light-induced DNA-binding protein - PubMed (nih.gov)]). There are crystal structures of NAM domains complexed with dsDNA (e.g. 3swm). SWI/SNF is involved in chromatin remodeling. However, electrostatic potential calculations do not show no clear face of potential that might signal specific binding to DNA (https://www.ncbi.nlm.nih.gov/pmc/articles/PMC7716260/). *abinA* (At3g16750) is an uncharacterized protein. Sequence searches bring up few homologs which seem taxonomically restricted to rosids. The protein has a negative net charge, making DNA binding unlikely. Nevertheless, there is striking conservation of a key interaction stabilizing the DNA recognition loop in the Gt1/Pac2 family (Figure 11).

In structural alignment view, click the *Show Stacked Sequence Logos* button to compare sequence conservation profiles between families. Convince yourself of the conservation of the DxxxW motif of the recognition loop (Figure 11).

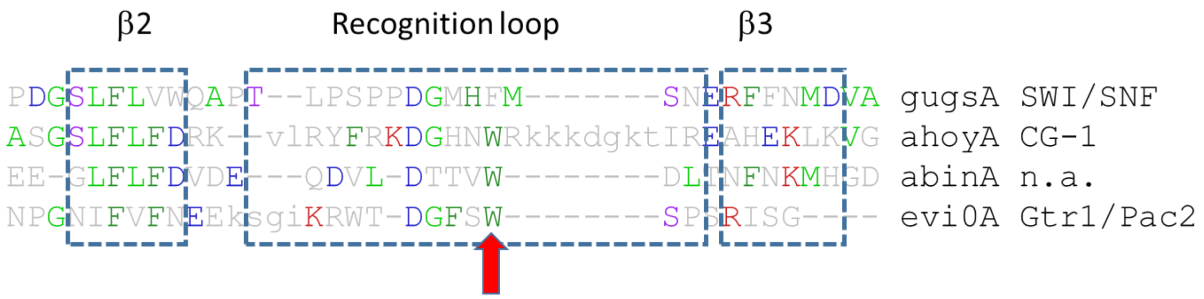

Figure 11. Structural alignment of the recognition loop. The red arrow points to W72, which is essential for DNA binding in the Gt1/Pac2 family ([Crystal structure of the WOPR-DNA complex and implications for Wor1 function in white-opaque switching of Candida albicans | Cell Research (nature.com)]). W72 forms a hydrogen bond to D68, stabilizing the loop. These residues are highly conserved in the other families.

6. Conclusion

Our analysis added three more Pfam families to the WRKY-GCM1 clan, namely CG-1, NAM, SWI/SNF, and a small family represented by At3g16750 (abinA). Despite strong structural similarity to sequence-specific DNA binding domains, electrostatic potential calculations (e.g. https://pdbj.org/eF-surf/top.do) do not support this function in the last two families. The starting point of this study, SWI/SNF, was accurately modelled from sequence by AlphaFold. CG-1 and abinA were modelled by AlphaFold and still lack experimental structures, making them interesting targets for structural genomics.

Box 1: Tips for discovery

1. Do a sequence search using SANSparallel. Terminate if found obvious homologs.
2. Do a PDB search using Dali server. Use PDB25 to remove redundancy.
3. Examine top-10 to top-20 hits. Define maximal common core guided by stacked structural aligment. Terminate if no compact, globular core can be found.
4. Check Pfam annotations of retained hits. Terminate if homology (family or clan) is known already.
   # At this point, we hypothesize a novel homologous relationship between protein/domain families.
5. Examine stacked Sequence Logos for sequence signals as possible evidence of homology.
6. Examine 3D superimpositions for peculiar structural features or conserved binding sites.
7. Do AF-DB searches using Dali server. Use Pfam annotations to select representative hits.
8. Add new Pfam families to the set of potential homologs, repeating steps 3-6.
   # At this point, we should have evidence of conserved, unifying features of potential superfamily.
9. Synthesis: check congruence of structural trees with functional diversification.
10. Prioritize targets for experimental validation.
